# Epigenetics in Kawasaki Disease

**DOI:** 10.3389/fped.2021.673294

**Published:** 2021-06-25

**Authors:** Kaushal Sharma, Pandiarajan Vignesh, Priyanka Srivastava, Jyoti Sharma, Himanshi Chaudhary, Sanjib Mondal, Anupriya Kaur, Harvinder Kaur, Surjit Singh

**Affiliations:** Department of Pediatrics, Advanced Pediatrics Centre, Post Graduate Institute of Medical Education and Research, Chandigarh, India

**Keywords:** Kawasaki disease, methylation, microRNA, long non-coding RNA, biomarker

## Abstract

Kawasaki disease (KD) is a common febrile multisystemic inflammatory illness in children that preferentially affects coronary arteries. Children with KD who develop coronary artery aneurysms have a life-long risk of premature coronary artery disease. Hypothesis of inherent predisposition to KD is supported by epidemiological evidence that suggests increased risk of development of disease in certain ethnicities and in children with a previous history of KD in siblings or parents. However, occurrence of cases in clusters, seasonal variation, and very low risk of recurrence suggests an acquired trigger (such as infections) for the development of illness. Epigenetic mechanisms that modulate gene expression can plausibly explain the link between genetic and acquired predisposing factors in KD. Analysis of epigenetic factors can also be used to derive biomarkers for diagnosis and prognostication in KD. Moreover, epigenetic mechanisms can also help in pharmacogenomics with the development of targeted therapies. In this review, we analysed the available literature on epigenetic factors such as methylation, micro-RNAs, and long non-coding RNAs in KD and discuss how these mechanisms can help us better understand the disease pathogenesis and advance the development of new biomarkers in KD.

## Introduction

Kawasaki disease (KD) is one of the common vasculitides that usually affects children below the age of 5. This condition is eponymously named after Dr. Tomisaku Kawasaki who described the disorder in 1967. KD is characterised by oral mucosal redness, conjunctival injection, rash, and swelling in hands and feet (1). Diagnosis of KD is based on a set of clinical features as enumerated by the American Heart Association (AHA). Main complication of KD is development of coronary arteries abnormalities (CAAs) that are seen in 15–20% of children with KD who do not receive treatment with intravenous immunoglobulin (IVIg) (2). Risk of development of CAAs reduces to 3–5% when therapy is initiated on time. However, around 15–25% of patients with KD do not respond to IVIg treatment and are said to have IVIg–resistant KD. These patients are at an increased risk of development of CAAs (3).

Incidence of KD in North East Asian countries is higher compared to countries in Europe and North America. Japan records the highest incidence of KD in world (308.0 per 100,000) (4), followed by South Korea (199.7 per 100,000 <5) (5) and Taiwan (82.8 per 100,000) (6). Several epidemiological and clinical reports have suggested that KD can be triggered by infectious agents including Mycoplasma, Chlamydia, Streptococci or Staphylococci (7–10) and viruses like adenovirus, Epstein-Barr virus, and parvovirus (11–13). Increased incidence of KD in certain population groups and increased risk of development of KD phenotypes in siblings of children who are affected with KD suggests involvement of genetic factors in susceptibility to KD.

Epigenetics is widely explored in most of complex heterogenic diseases like KD where contribution of both genetic and environmental factors is equally shared (14–17). Genetic predisposition of complex disease phenotypes has been associated with substantial contribution of dysregulated epigenetic factors. Moreover, it has also been established that interplay of genetic, epigenetic, and environmental factors in several rheumatological illnesses play a crucial role in disease severity and treatment responses. Epigenetic factors can also potentially explain the phenotypic heterogeneity and differential treatment response.

Complex genetic architecture of KD suggests contribution of several innate and adaptive immune responses, extracellular matrix (ECM) components and proteins regulating its remodelling, and angiogenic proteins (14–17). Apart from widely explored genetic network in KD, a well-defined biomarker for diagnosis and prognostication has not yet been established. Exploration of epigenetic risk factors in KD, hitherto unexplored until date, could pave way for identification of potential biomarkers in future and help in advancement of precision medicine.

Advancement in molecular technologies has led to identification of several post-translational regulatory factors such as miRNA and long non-coding RNAs (lncRNA). Recently, involvement of gut microbiota and associated immune modulation has also been implicated in the pathogenesis of KD. In this review, we aim to focus on epigenetic studies that have been carried out in KD, discuss their potential contribution to our understanding of the disease pathogenesis, and their role in the development of biomarkers.

## Search Strategy

We carried out literature search through PubMed/ Medline and Embase databases using the keywords in different combinations: Epigenetics, genetics, methylation, microRNAs, long non-coding RNA, Kawasaki disease. Original articles and reviews related to genetic and epigenetic studies in KD published in English language until 20 February 2021 were retrieved and analysed.

## Pathophysiology and Genetic-Architecture of KD

Pathogenesis of KD involves several immunological and non-immunological genes. Activation of endothelial cells under the influence of S100 and HMGB1 in KD can be regulated through the action of pathogen-associated molecular patterns (PAMPs) and danger-associated molecular patterns (DAMPs) suggesting involvement of innate immunity in the KD (18–23). Infiltration of CD14+ monocyte/macrophages in the coronary walls has also been noted in patients with KD (23). Identification of genetic variants of ITPKC, CASP3, CD40, and ORAI in KD signifies the involvement of T-cell activation and differentiation (24). Recent report from Onouchi et al. has demonstrated impaired development and function of B-cell (rs2254546 for FAM167A-BLK; src family B lymphoid tyrosine kinase) and augmented activation of T-cells (rs4813003 CD40) in KD. These results have also been supported by the studies carried out in Taiwanese and Han Chinese population groups (25–29). Moreover, six SNPs located in variable region of heavy chain of immunoglobulin gene were also shown to have a strong association with KD in Taiwanese population (22). Association between HLA genetic variants and formation of coronary artery lesions (CALs) in KD has been established in several ethnicities including Chinese (HLA-A, HLAB, HLADR), Japanese (e.g., Bw54, Bw22J, Bw22J2), and Caucasians (Bw51, Bw44, DRB3, DRB1^*^301, DRB1, DQA1, DQB1, DPB1) (30–36). All these studies suggest pathological significance of both adaptive and innate immunity in KD.

Wang et al. have studied the role of Th1/Th2 secreted cytokines in patients with KD. Levels of IL-6, IL-10, TNFα, and IFNγ were significantly elevated in acute stages of KD and all, except TNFα levels, decreased after IVIg treatment. On subgroup analysis, levels of TNFα were found to decrease in IVIg-responder group, and in patients with KD without CAAs. Higher TNFα levels were observed in IVIg resistant KD and in patients with CAAs. Results have also showed that patients with KD and CAAs exhibited significant elevation in levels of IL-4, IL-6, IL-10, and IFNγ. After IVIg therapy, IL-6 and IL-10 levels were significantly higher in IVIg-resistant group when compared with IVIg-responder group (37).

Higher proportion of Th17 (helper type 17) cells with concordant increased expression of cytokines IL-17, IL-6, IL-23, and transcription factors IL-17A/F and ROR-γt were observed in acute phase of KD. IVIg-resistant patients had higher proportion of Th17 cells compared to IVIg-responsive patients. These studies have also showed the pathological importance of regulatory T cells which were found to be decreased in children with acute KD (38). Moreover, decrease proportion of CD4 + CD25 + FOXP3 + Treg cells have been associated with IVIg resistance in KD (39, 40). In another study, significantly decreased levels of both IL-6 and IL-17A, and elevated levels of FOXP3, were reported in patients with KD after IVIg treatment. Increased levels of IL-17A and IL-10 levels were found to be associated with an IVIg resistant phenotype of KD (41). A decreased T-cell proliferative response (TCR/CD3 mediated) has also been observed in KD and this may affect the production of pro-inflammatory cytokines (24, 29, 38, 39, 42).

Extracellular matrix (ECM) proteins (including collagen and integrin proteins) have been widely studied in KD. Abnormalities of ECM remodelling have been linked with development of coronary artery lesions (CALs) in KD. MMPs play a vital role in ECM remodelling, neointimal proliferation, and initiation of angiogenesis (43–46). They also have an important role in maintaining the integrity of coronary vascular wall structure and function. MMPs also mediate macrophage invasion and tissue destruction. Differential expression of MMPs at systemic and micro-environmental levels has been documented in KD. Abolition of MMP-9 activity stimulates the inflammatory responses after exposure to *Lactobacillus casei* cell wall extract (LCWE) in a mice model of KD (47).

The sub-acute and chronic vasculitis in KD originates from smooth muscle cell-derived myofibroblasts under the differential expression of collagen type I, α1 (COL1A1), MMP7, and integrins α4 and αM (ITGA4, ITGAM). Study on LCWE induced murine model of KD has emphasised the augmented TGF-β signalling and proteolytic activity of elastin in wall of coronary arteries. Studies have also demonstrated the reduction of plasminogen activator inhibitor-1 (PAI-1, proteolytic inhibitor) as well as increased levels of MMP9 (matrix metalloproteinase-9) after administration of TGF-β inhibitor (48).

Interestingly, ECM components have also been shown to have immune-modulatory effects. Denatured collagen type 1 has showed the ability to adhere with scavenger receptors of innate immunity and found to modulate the secretion of MMP9 from macrophages (49, 50). Disintegrated ECM can also serve as catalyst for coronary aneurysm formation in KD with enhanced transmural infiltration of activated T-cells. Moreover, the infiltration of macrophages in the adventitious layer of coronary arteries has also been observed in patients with KD (51). Altered expression of adhesion molecules like E-selectin or VCAM-1 (vascular cell adhesion molecule-1) could also facilitate the infiltration of inflammatory cells at the site of inflammation in KD (52). Proteins involved in TGF-β pathway along with over-expression of IL-6 and IL-17 in infiltrating monocytes have been implicated in development of thickened intima of affected coronary arteries in patients with KD. Activated CD8+ (HLA-DR positive) and FOXP3+Treg cells were also observed in coronary arteries of patient group implicating the role of effector T cells and TGF-β signalling in KD (53).

## Epigenetic Regulation in KD

Involvement of a wide spectrum of immunological and non-immunological factors indicates heterogenic genetic architecture that contributes to pathogenesis of KD. In addition to genetic factors, several environmental factors including geographical distribution of population and infections could also equally contribute to KD. Recently, epigenetic regulation of genes involved in the pathogenesis of KD has also been studied. Epigenetic modification can occur by several ways including covalent modification of cytosine (e.g., methylation by DNA methyltransferases), post-transcriptional modification of histone protein (e.g., acetylation, phosphorylation, methylation, citrullination, ubiquitylation, ribosylation, and sumoylation), and RNA based regulation of transcriptional machinery (54). Exploration of epigenetics especially in complex disease phenotypes like KD is warranted wherein both genetic (even genetic interactions) and environmental factors contribute to disease pathogenesis and can influence the treatment response.

### Methylation and KD

Methylation is the most widely studied epigenetic factor in KD. Mechanism of methylation is regulated by a set of DNA methyltransferase (DNMTs) enzymes along with other assisting proteins. Covalent binding of a methyl group (-CH3) to CpG island of the genome (especially on promoter region) can modulate the expression of genes and subsequently influence protein expression without altering genetic sequence.

Huang et al. have shown that expression levels of DNMT1 and DNMT3A were significantly lower in patients with KD compared to normal controls. Authors concluded that transient DNA methylation occurs during acute stage of KD (55). Another study by Li et al. has analysed the changes in DNA methylation in patients with KD before and after IVIg administration. Treatment with IVIg has been shown to alter methylation mainly by hypomethylating CpG markers. Pathway enrichment analysis showed that genes regulated by altered methylation after IVIg administration are associated with inflammatory response (56). Chen et al. (57) studied difference in methylation between patients with KD and control population. Authors observed that 97% of the CpG regions that had more than 20% difference of methylation between KD patients and controls were hypomethylated (57). Most of the differentially methylated regions appear to be enriched in genes involved in inflammatory response, innate immunity, and chemokine signalling pathways. Epigenetic changes in patients of KD have been observed in genes such as FCGR2A, matrix metalloproteinases (MMPs), toll-like receptors (TLRs), NOD-like receptors, HAMP giving insight in pathophysiology of KD.

Toll-like receptors (TLRs) are involved in triggering a proinflamatory cytokine cascade in response to several antigens including bacterial lipopolysaccharide (LPS). TLRs widely recognise signature patterns presented on bacteria (lipopolysaccharide) and viruses (dsDNA/RNA, ssDNA/RNA), mediate inflammatory responses and biosynthesis of cytokines through the activation of protein kinases (e.g., IRAK1/4, TBK, and IKKi, etc.). Patients with KD were shown to have elevated mRNA expression of TLRs except for TLR3 and TLR7 in the acute stage. However, methylation patterns of CpG sites on TLR1, 2, 4, 6, 8, and 9 were found to be lower in acute stages of KD when compared to normal controls. These patterns increased significantly after IVIg treatment (58). Stimulation of TLR2 and TLR4 with LPS has been shown to modulate the expression of IL-2, IL-6, IL-10, MCP-1, TNF-α, and INF-β in mice models of KD (59, 60). This observation provides preliminary evidence that KD is triggered by a bacterial agent.

Polarisation of macrophages from M1 to M2 has been noted in acute stages of KD. Recently, transcriptomic profiling using GeneChip array in patients with acute phase of KD has showed elevated expression of *TLR2* and *IL2RA* (M1 macrophage) in addition to markers associated with M2 macrophage (e.g., *MS4A4A, MS4A6A, TLR1, TLR8, TLR5, CD36, CCR2*, and *ARG1*). Authors have also noted hypomethylation of the promoter region of respective genes (17). IVIg treatment given to these patients has further enhanced the expression pattern of these genes by enriching the hypomethylation status. Trans-endothelial movement of leukocytes at site of inflammation is regulated by “S100A genes.” Increase in expression of these genes result in enhanced trans-endothelial migration of leukocytes. Hypomethylation and hypermethylation of S100A genes has been noted in acute and convalescent phases of KD, respectively (16). Abnormal neutrophil activation has also been associated with KD. Enhanced expression of neutrophil activation marker CD177 with abnormal methylation patterns has been observed in patients with KD. While the levels of CD177 were found to be decreased in patients with IVIg-responsive illness, the levels were increased in patients with IVIg-resistant KD (61).

NOD-like receptors (NLRs) are cytoplasmic sensors for endogenous or exogenous damaging molecules. They form part of the inflammasome structure, and downstream signalling through NLRs activates caspase (CASP) protein (especially CASP1). Activation of CASP1 has been noted in acute stages of KD, which further facilitates the synthesis of pro-inflammatory cytokines like IL-β1 and IL-18 (62–66). NLRC4 (Nucleotide-binding protein) is a key molecule to activate CASP1 protease to ultimately direct the synthesis of the IL-β1 from its precursor IL-β1 (pre-IL-β1). Experimental studies in mice have shown that caspase 1 and IL1-β are required for development of CAL in KD (67). Hypomethylation and hypermethylation of CpG (promoter region) have been observed in *NLRC4* and *NLRP12*, respectively, in patients with KD. Subsequently, this resulted in increased expression of NLRC4 and IL-β1, and deceased expression of *NLRP12*, which were further found to be associated with formation of CAL in KD (15).

Fc receptors [Immuno-receptor tyrosine-based activation motif (ITAM)-associated receptor family] in the immunocytes bind to antibody or antigen-antibody complexes and help in regulation of opsonization, phagocytosis, degranulation, and cytokine biosynthesis (68, 69). Changes in expression levels or altered function of Fc receptors have been implicated in the immunopathogenesis of several rheumatological diseases including KD (70–72). Genome-wide association studies (GWAS) had identified *FCGR2A* gene as a susceptibility gene for KD. *FCGR2A* codes for the immunoglobulin IgG (gamma Fc region receptor II-a) receptor that is widely expressed in activated immune cells. Studies had shown that methylation level of this gene affects the binding of the IgG2 to the receptor (73). A recent GWAS conducted on the Han Chinese population has identified 10 SNPs that confer genetic susceptibility in KD patients. One of the SNPs identified in this study was *FCGR2A* (rs1801274) (74). Epigenetic regulation of *FCGR2A* with 15.54% hypomethylation pattern has also been demonstrated in KD patients in comparison to controls as reported by Kuo et al. (75). Significantly higher hypomethylation was also documented in patients with IVIg resistance compared to patients who responded to first dose of IVIg. Variations in gene copy numbers of FcγR2C and FcγR3B have also been shown to be associated with predisposition to KD and IVIg responsiveness (76). Chang et al. showed that methylation of FCER1A was lower in patients with KD compared to controls (77). This provides evidence for the role of IgG receptors in the pathogenesis of KD (Table 1).

**Table 1:** Tabular representation of studies related to regulation of genes by methylation, downstream cellular mechanism and implication in KD.

**Pathological contribution**	**Comparative groups**	**Samples**	**Approached used**	**Methylation pattern**	**Gene regulated**	**Mechanistic contribution**	**References**
Methylation regulation	KD and controls	Blood	Affymetrix GeneChip® Human Transcriptome Array 2.0	Hypo- and hyper-methylation of DNA methyltransferases	Decreased expression of DNMT1 and DNMT3A; TET2 higher	Transient hypomethylation in acute KD	(55)
Regulation of innate immunity	18 healthy controls, and 18 febrile controls	Blood	Affymetrix GeneChip® Human Transcriptome Array 2.0 and Illumina HumanMethylation450 BeadChip	Hypo-methylation	TLR1, TLR2, TLR 4, TLR 6, TLR 8, and TLR 9	Bacterial inflammatory response may trigger KD	(58)
	Taiwan population						
	KD (18) and controls (18)	Blood	GeneChip Human Transcriptome Array 2.0 and Illumina HumanMethylation450 BeadChip	Hypomethyaltion of M1 and M2 macrophages markers	M1 markers (TLR2, IL2RA) and M2 markers (ARG1, CCR2, TLR1, TLR8, TLR5, MS4A6A, CD36, and MS4A4A)	Macrophage polarisation in KD	(17)
	Taiwan population						
	KD and Controls	Blood	DNA methylation (M450K)	Hyper-methylation of S100 genes	repressing S100A genes' expressions	Impaired transendothelial migration of neutrophil	(16)
	Taiwan population						
Methylation of inflammatory genes	KD (18) and controls (36)	Blood	Illumina HumanMethylation 450 BeadChip and Affymetrix GeneChip® Human Transcriptome Array 2.0. A	Hypermethylation of NLRC4, NLRP12, and IL-1β	NLRC4, NLRP12, and IL-1β higher	Reversed the expression of genes after IVIG treatment; NLRP12 associated with CAL	(15)
	Taiwan population						
	7 acute phase KD (KD1); 7 KD patients 3 weeks after IVIG treatment (KD3) and 4 febrile controls	Blood	Illumina HumanMethylation27 BeadChip	Altered methylation of 3,249 CpG markers KD1 vs. febrile control; 5,438 CpG markers in KD3 vs. febrile control and 5,353 CpG markers in KD3 vs. KD1.	Genes involved in hematopoietic lineage, chemokine signalling and inflammatory cascade.	Hematopoietic cell lineageChemokine signalling pathwayCytokine-cytokine receptor interactionJak-STAT signalling pathway	(56)
Regulation of adaptive immunity	KD and controls	Blood	HumanMethylation27 BeadChip	15.54% less methylation of FCGR2A	CD40, BLK, and FCGR2A	FCGR2A in susceptibility to KD and IVIG resistance	(75)
	Taiwan population						
	18 healthy controls, and 18 febrile controls	Blood	Pyrosequencing	Methylation pattern	Decrease levels of FCER1A and FCER2; increase levels of FCER1G after IVIG; lower FCMR rose after IVIG treatment	Implication of IgA, IgE, IgG, and IgM receptors in KD	(77)
	Taiwan population						
Methylation in ECM remodelling	24 KD patients and 24 non-KD controls	Blood	HumanMethylation450 BeadChips	3,096 (out of 3,193) CpG loci revealed hypomehtylation, with only 3% being hypermethylated	NFAT, ETS1 (Avian), RUNX3 and RARG and β-catenin; decrease CTNNB1 levels with CAL	Crucial role of β-catenin in CAA formation and cardiac function in KD	(57)
	18 healthy controls, and 18 febrile controls	Blood	Illumina HumanMethylation450 BeadChip and Affymetrix GeneChip® Human Transcriptome Array 2.0	Hypomethyaltion of MMP-9	MMP-8,−9, and−25 levels high	MMP-9 in KD with CAL	(14)
	Taiwan population						
	31 KD and 14 healthy controls	Blood	MethylTarget sequencing	hypomethylation	CTRP1	methylation levels of CTRP1 promoter could contribute in CAA development	(78)
	24 KD patients and 24 non-KD controls	Blood	HumanMethylation450 BeadChips	Total methylation difference at 3193 CpG sites between KD and controls which consists 3,096 CpG loci with hypomehtylation and 3% hypermethylated regions.	Nuclear factor of activated T-cells 1 (NFAT1), v-ets avian erythroblastosis virus E26 oncogene homologue 1, runt related transcription factor 3, and retinoic acid receptor gamma, and activator β-catenin.	Pathological implication of β-catenin in KD.	(57)
	KD patients (*n* = 18) (pre-post-after 3 week of IVIg) and 18 febrile controls	Blood	Illumina HumanMethylation450 BeadChip	Hypomethylation of HAMP promoter	Hypomethylation of HAMP promoter and decreased levels of hepcidin which were restored after IVIg treatment	Implication of iron transportation in KD	(79)

Association of epigenetic regulation of ECM remodelling with CAA formation in KD has not been much explored. MMPs have an important role in maintaining coronary vascular wall structure and function. They also mediate macrophage invasion and tissue destruction. Kuo et al. found significant differential expression of MMPs 8, 9, and 25 in patients with KD in comparison to healthy and febrile controls (14). Methylation levels of these MMPs were significantly lower in KD patients. MMP-9 showed significant hypermethylation after IVIg treatment in patients with KD. IVIg resistant patients with CAL associated KD have exhibited higher expression of MMP9. Studies have demonstrated the association of various cytokines such as TNF-alpha and IFN-γ with MMPs suggesting that the MMPs can serve as KD biomarkers (14) (Table 1).

Similarly, collagen binding protein CTRP1 (C1q/tumour necrosis factor-related protein-1) has also demonstrated reduction in CpG methylation at multiple sites near promoter region (nucleotide sites at 34, 51, 69, 79, 176, and 206) whereas hypermethylation pattern was observed at the promoter sites 69 and 154 in CAA group (coronary artery aneurysms). Results have suggested the CpG methylation of CTRP1 as prominent marker to distinguish KD with and without CAA (74, 78). Huang et al. (79) in a cohort of patients with KD have also assessed the methylation and functional aspects of promoter region of *HAMP* that codes for hepcidin (regulate iron transportation). Hypomethylation of *HAMP* (at CpG sites cg23677000 and cg04085447) with subsequent elevation in hepcidin levels has been noted in patients with KD. Levels of hepcidin decreased after IVIg treatment in patient group (79). This study suggests that iron transportation is probably involved in ECM remodelling and CAA formation in KD.

Clusters of few hypermethylated regions have also been shown to positively correlate with KD susceptibility and CALs in KD. The biological context of these differentially methylated regions (DMRs) has been obtained by network and pathway analysis studies. One such analysis identified a network of genes in the β-catenin pathway which plays an important role in cardiac repair (80). Identified gene network consists of transcription activator β-catenin (CTNNB1- a core regulator in the network) along with associated transcription factors (NFATC1, ETS1, RUNX3, and RARG), correlated regulators CDC25B and PDCD1, and the related effectors LTA, BTLA, and CD247. mRNA levels of five genes (CTNNB1, RUNX3, ETS1, NFATC1, CD247) were considerably lower in KD patients as compared to controls, while expression levels of β-catenin (CTNNB1) were significantly lower in KD patients with CAL when compared to non-CAL patients. Decreased CTNNB1 expression in monocyte cell line has been shown to increase expression of CD40 and CD40L in human coronary artery endothelial cells (57).

### Role of microRNA in Kawasaki Disease

MicroRNAs (miRNAs) are endogenous, small (18–25 nucleotides long) non-coding, single stranded RNAs which play an important role in controlling mRNA translation. Non-coding RNAs are transcribed but not translated into proteins, and act as an epigenetic mechanism to regulate gene expression. They regulate the gene expression after transcription by binding the translation section. This results in either mRNA degradation or translational inhibition (81, 82). miRNAs target the 3′ untranslated region (3′UTR) of their target mRNA molecule and control their stability and protein interactions (83). These are highly conserved, and their expression is time specific. miRNA expression is controlled by other epigenetic mechanisms and itself controls these mechanisms as an “epigenetics-miRNA regulatory circuit” that, when perturbed, can contribute to disease pathogenesis (84).

In humans, miRNAs are thought to play an important role in regulation of coding genes. miRNAs are considered to be a useful biomarker for many pathogenic states, aiding in both diagnosis and prognosis These are also involved in several cellular processes such as cell proliferation, apoptosis, migration, invasion, and stress response (85–87). Unique expression patterns of miRNAs may be used as a novel, non-invasive biomarker for disease diagnosis (88). miRNAs have been reported in tissues and also known to be released in peripheral blood. These circulating miRNAs have been found to be associated with a specific pathophysiological state (89). Therefore, peripheral serum microRNAs are widely used as diagnostic and therapeutic biomarkers for a variety of diseases. Aberrant miRNAs expression has been reported to be associated with several diseases including cancers, cardiovascular diseases, inflammatory conditions and immune regulation (90–93).

Increasing evidence suggests that miRNAs have a role in the pathogenesis of several human diseases, including KD (94). Previous reports on circulating miRNAs expression in KD are mentioned in Table 2.

**Table 2:** Regulation of various genetic and cellular networks involved in KD by miRNAs and lncRNAs.

**Broad function**	**References**	**miRNA source**	**Comparative group**	**Approach used**	**miRNA**	**miRNA expression**	**Disease contribution**
Regulation of TGF-β pathway	(95)	Whole blood and Plasma	12 KD patients; (during acute and convalescence)	High throughput sequencing	miR-143, 199b-5p,-618,-223,-145, and 145	↑	Regulates TGF-β signalling
			12 febrile controls				
	(96)	HUVEC and C2C12	Cell culture of HUVEC and C2C12myoblasts	qRT-PCR	miR-200c	↑	Regulates TGF-β and oxidative stress pathways
	(97)	Serum	12 KD patients and 6 afebrile controls	miRNA microarray	miR-200c, miR-371-p	↑	Regulates TGF-β and oxidative stress pathways
	(94)	Serum	10 KD patients and 10 febrile childhood controls with a variety of infectious/inflammatory conditions	Real Time PCR	miR-145	No significant alteration	
	(98)	Serum	KD: 84	Real Time PCR	miRNA-210-3p, miRNA-184, miRNA-19a-39	↑	
			Non KD febrile: 29				
Regulation of inflammatory responses	(99)	Blood	KD: 50 CAAs: 5 Non KD febrile: 25 Healthy: 25	microRNA array	MicroRNA-145-5p ↑ microRNA-320a ↑		Specific miRNAs encapsulated in EMPs may modulate the secretion of inflammatory cytokines from monocytes/macrophages.
	(100)	Serum	102 KD patients and 80 healthy controls	qRT PCR	miR-200c, miR-371-5p	↓	Involved in pro-inflammatory response
	(101)	Serum	5 KD and 5 KD with three week follow up after IVIg and 5 controls	Microarray	65 differentially expressed miRNAs were estimated in which miR-328, miR-575, miR-134, miR-671-5p were prominently involved in negative regulation of inflammatory genes	Differentially expressed	Regulation of pro/inflammatory genes expression
Regulation of adaptive immunity	(102)	CD19+ B cells	Acute KD patients and healthy control children	Real Time PCR	miR-27a-3p	↑	Pro-inflammatory genes (IL-10 and TNF-1lfa), inhibit B10 cell function.
	(103)	CD4+CD25+ T cells	33 Acute KD and 14 controls	Real Time PCR	miR-21	↓	Decreased Treg differentiation and function
					miR-155	↓	
					miR-31	↑	
	(104)	Platelets	Acute KD patients and other febrile patients	high-throughput miRNA sequencing	miR-222-3p	↑	Immune-related signalling pathways.
Endothelial cell integrity and functions	(85)	PBMCs	23 KD and 12 controls	Reverse-transcriptase PCR (RT-PCR) and enzyme-linked immunosorbent assay (ELISA)	MIR93	Increased miR-93 in patients responding to IVIG; inverse correlation with VEGFA mRNA expression	Regulate expression of VEGF-A
	(105)	Plasma	6 acute KD patients and 6 healthy control children	Microarray	hsa-let-7b-5p, hsa-miR-223-3p, hsa-miR-4485, hsa-miR-4644, hsa-miR-4800-5p, hsa-miR-6510-5p, hsa-miR-765	↑	
					hsa-miR-33b-3p, hsa-miR-4443, hsa-miR-4515	↓	
	(106)	Plasma	30 acute KD 30 convalescent KD 32 healthy controls	qRT-PCR	hsa-miR-125a-5p, hsa-miR-133a, miR-148a, miR-199b-5p, miR-223, miR-330-3p, miR-483-5p, miR-671-3p, miR-744, miR-885-5p, miR-7	↑ MIR125A	Inhibition of MKK7 expression which promotes caspase-3 expression and apoptosis in endothelial cells
	(107)	Serum exosomes	20 healthy individuals, 20 KD patients before IVIG treatment and 20 KD patients after IVIG treatment.	High-throughput microarray technologies, two-stage real-time quantitative PCR	MIR1246/MIR4436B1 and MIR197/MIR671	MiRNA pairs that, when combined, can differentiate KD patients from controls and non-KD febrile cases	miR-197 is predictive of death in symptomatic coronary artery disease and miR-1246 is a biomarker for diastolic dysfunction
	(108)	Total leukocytes	37 Fever Controls and 31 KD	NGS/qPCR	MIR223	↑ In KD patient serum especially those with coronary artery lesions; identified as part of KD diagnostic miRNA panel in total leukocytes	Released by bone-marrow derived blood cells into serum; promotes apoptosis in endothelial cells by targeting IGF1R and suppresses cell proliferation
	(109)	Serum	21 acute KD children, 25 healthy controls, 17 febrile children	RT-qPCR	miR-186	↑	miR-186 has an essential role in endothelial cell apoptosis by activating the MAPK pathway through targeting the SMAd6 gene.
			11 convalescent KD children				
	(110)	Serum	45 KD acute and paired convalescent serum specimens 30 febrile children and 30 healthy controls	qRT PCR	miR-92a-3p	↑	Arterial endothelial dysfunction of KD.
	(111)	Serum	Acute KD patients and healthy control children	RT-qPCR	miR-197-3p	↑	miR-197-3p modifies cell behaviours of proliferation, apoptosis and migration by targeting IGF1R and BCL2 in KD
	(110)	Serum	KD patients and healthy control	RT-qPCR	miR-27b	↑	Regulate proliferation and migration of endothelial cells under the function of Smad7 and TGF-β pathway
	(109)	Serum	KD patients and healthy control	RT-qPCR	miR-186	↑	Induces endothelial cells apoptosis by inhibiting the action of SMAD6 *via* MAPK pathway.
**Long non-coding RNA in KD**							
Inflammatory response	(112)	Blood	37 KD patients and Febrile controls	Real Time PCR	XLOC_006277	↑	Increase in expression of MMP-8 and MMP-9 along with CD177 associated with CAL indicating neutrophil activation in KD
	(113)	Blood	KD patients	Microarray RNA-Seq	linc1992 THRIL	Regulate TNF-α	linc1992 is shown to induce TNF-α expression and knockdown of this lncRNA resulted in dysregulated innate immune response
Endothelial cell integrity and function	(114)	Blood	Human umbilical vascular endothelial cells (HUVECs)—vascular inflammation model for KD	Real Time PCR	Pregnancy-induced non-coding RNA	↑ in TNF-α	Inhibit HUVECs proliferation by regulating the action of TNF-α

Studies have shown that there were differential expressions of microRNAs in children with KD and during the acute stage of KD, miR-143, miR-199b-5p, miR-618, miR-223, and miR-145 were significantly higher (95, 97, 109, 110). Another study showed that serum miR-200c and miR-371-5p were significantly higher in KD patients as compared to healthy controls. These miRNAs may have an important role in disease pathogenesis and can be potential biomarkers for KD (97). Both exosomal and serum miRNAs e.g., miR-1246, miR-4436b-5p and miR-197-3p, and miR-671-5p have been studied for its role as biomarkers in KD (98, 107, 108). Zhang et al. studied 102 KD patients and showed that serum miR-200c and miR-371-5p were significantly elevated in sera in acute stages of KD. The levels were higher in the group with IVIG resistance who required further plasma therapy (100). They also showed that levels decreased significantly following therapy. In another study by Zhang et al., serum exosomal miR-328, miR-575, miR-134 and miR-671-5p have been shown to act as potential biomarkers for the diagnosis of KD and the prediction of outcomes of the IVIg therapy by influencing the expression of inflammatory genes (101). Wang et al. showed that hsa-let-7b-5p and hsa-miR-223-3p were slightly downregulated, while miR-200c, miR-197-3p and miR-671-5p was upregulated in KD (104).

Encapsulated miRNA-145-5p/miRNA-320a in endothelial particles has been shown to modulate inflammatory response and progression of vasculitis in patients with KD (99). Similarly, enhanced levels of miR-27a have been shown to induce inflammatory responses mediated by monocyte derived TNF-α synthesis in patients with KD. Results have also revealed negative regulation of IL-10 secretion from B cells under action of TNF-α (102). Many other miRNAs including miR-200c, miRNA-145-5p, miRNA-320a, and MiR-197-3p have also been shown to regulate the proliferation, migration and apoptosis/senescence of endothelial cells by moderating the action of various downstream molecules e.g. ZEB1 (Zinc finger E-box-binding homeobox 1, inhibit T cell specific IL2 expression), IGF1R (insulin-like growth factor 1) and BCL2 (96, 111).

As exogenous miR-223 has been shown to exert biological effects on endothelial cell (EC) functions *via* its target genes such as IGF1R (115). A study conducted by Chu *et al* assessed miRNAs profiles in patients with KD and found that miR-223 was significantly elevated in these patients (115). They also postulated that KD-induced EC injuries were related to increased miR-223 because they were inhibited by miR-223 knockdown. Increased miR-223 in ECs could work as a novel endocrine genetic signal and participate in vascular injury of KD. Li et al. have shown that 18 miRNAs were differentially expressed in plasma of patients with KD when compared with healthy controls (106). miR-125a-5p was significantly increased in plasma of KD patients and has been considered to play a role in pathogenesis of KD by regulating target gene MKK7 to induce apoptosis in vascular endothelial cells. Jia et al. have suggested a set of 4 miRNAs which could distinguish KD patients from other febrile patients as well as from healthy individuals in a single pass (106). They identified 4-miRNA set [namely, CT(miR-1246)-CT(miR-4436b-5p) and CT(miR-197-3p)-CT(miR-671-5p)] in 79 samples from two hospitals and proposed them as candidate diagnostic biomarkers for KD. Another study uncovered 7 miRNAs that were significantly upregulated (hsa-let-7b-5p, hsa-miR-223-3p, hsa-miR-4485, hsa-miR-4644, hsa-miR-4800-5p, hsamiR-6510-5p, and hsa-miR-765) and 3 that were significantly downregulated (hsa-miR-33b-3p, hsa-miR-4443, and hsa-miR-4515) in plasma of acute KD compared with healthy controls (105). MiR-93 may regulate vascular endothelial growth factor A (VEGFA) of circulating peripheral blood mononuclear cells in children with acute KD (85).

Previous studies have shown that in absence of specific miRNAs, forkhead box protein 3 (FOXP3)+ regulatory T cells (Treg) develop but fail to maintain immune homeostasis. Ni et al. (103) demonstrated that FoxP3 mRNA levels were primarily affected by the miR-155/SOCS1 (suppressors of cytokine signalling) and miR-31 signalling pathways. These results suggest that decrease in FoxP3+ Treg might be associated with decreased expression of miR-155, leading to aberrant SOCS1/STAT-5 signalling and overexpression of miR-31 in patients with acute KD. IVIg treatment may rescue Treg number and function by regulating miR-155 and miR-31 expression.

### Role of Long Non-coding RNA in Kawasaki Disease

Long non-coding RNAs (lncRNAs) are a large class of ncRNAs (longer than 200 nucleotides in length) involved in many diverse biological processes through the regulation of gene transcription (116, 117). Over 27,000 lncRNAs have been predicted/annotated in the human genome and there is emerging evidence indicating their functional diversity and relevance in regulating human disease and development (117–119). Studies on lncRNA in KD are only handful.

Ko et al. (112) from China investigated changes in expression of lncRNAs in 37 patients with KD. Long non-coding RNA profiling revealed that the transcript XLOC_006277 was overrepresented in patients with acute KD and decreased after IVIg in IVIg-responsive patients. Notably, higher levels of XLOC_006277 transcript were found in patients with KD who later developed CAAs (112) (Table 2).

Many lncRNAs can enhance the inflammatory response by increasing the transcription of pro-inflammatory cytokines or other inflammatory target genes or by enhancing inflammatory signals. THRIL (TNF- and hnRNPL-related immunoregulatory lncRNA) is one of the many lncRNAs induced after TLR2 activation (113). CXCL10, one of the genes found to be regulated by THRIL, is up-regulated in KD patients in the acute phase and has been identified as a possible biomarker of the disease (120). In 17 patients with KD, linc1992/THRIL expression was lower during acute phase of disease when TNFα levels are elevated. Authors suggested linc1992/THRIL could be a new biomarker for immune activation in KD (113). In a study by Jiang et al., pregnancy-induced non-coding RNA (PINC) was shown to have an elevated expression in human umbilical vascular endothelial cells that are treated with TNF-α. The same study showed that PINC is involved in regulation of TNF-α induced vascular endothelial cell apoptosis (114).

In summary, lncRNAs are induced in response to inflammation and regulates gene transcription during the inflammatory response. Though there is limited literature available, yet there is compelling evidence that KD is associated with changes in expression of lncRNAs. However, further research is required to get an insight into the biological function and role of lncRNAs in KD for the development of new strategies for its treatment and as biomarker assessment.

## Conclusion

Epigenetic studies have unravelled several new biomarkers for diagnosis and disease prognostication in KD. Majority of these are single-centre studies based on limited number of patients. These biomarkers, however, would need validation on larger cohorts and in different population groups before the results can be translated to clinical practise. Techniques and expertise required for carrying out epigenetic tests are demanding and the results may not be available quickly easily for bedside management of patients. However, with rapid decline in costs of genetic sequencing technology in recent years, it is possible such tests would be more utilised in future.

## Author Contributions

KS, PV, PS, JS, AK, and HK preparation of manuscript. PV, PS, and SS editing and critical review of manuscript. HC and SM performed experiments and literature search. All authors approved the final version of manuscript.

## Conflict of Interest

The authors declare that the research was conducted in the absence of any commercial or financial relationships that could be construed as a potential conflict of interest.
